# Accumulation of recalcitrant dissolved organic carbon during cyanobacterial blooms in Meiliang Bay, Lake Taihu: insights into the microbial carbon pump

**DOI:** 10.3389/fmicb.2026.1753025

**Published:** 2026-02-13

**Authors:** Xiaohan Wu, Xiaogang Chen, Dan Wu, Fenfen Zhang, Jinzhou Du

**Affiliations:** 1 State Key Laboratory of Estuarine and Coastal Research, East China Normal University, Shanghai, China; 2 Key Laboratory of Coastal Environment and Resources of Zhejiang Province, School of Engineering, Westlake University, Hangzhou, China

**Keywords:** cyanobacterial bloom, eutrophic lake, recalcitrant dissolved organic carbon, fluorescent dissolved organic matter, bacterial community, NMR

## Abstract

**Introduction:**

Cyanobacterial blooms are increasing in frequency, intensity, and duration in both freshwater and marine environments, potentially enhancing carbon sequestration by producing recalcitrant dissolved organic carbon (RDOC).

**Methods:**

We conducted monthly analyses of dissolved organic matter (DOM) composition and bacterial community dynamics in Lake Taihu (Meiliang Bay), China, integrating fluorescence DOM and ^¹^H NMR to quantify carboxyl-rich alicyclic molecules (CRAM) as a molecular proxy for RDOC.

**Results:**

Estimated CRAM increased from 51.86 ± 11.22 μM C in the non-bloom period to 60.80 ± 8.21 μM C during blooms (~17% higher). The annual average RDOC was 62.93 ± 10.66 μM C, accounting for ~16% of the total DOC. Bacterial community analysis revealed that labile DOC was actively metabolized and transformed into more recalcitrant compounds through microbial carbon pump mechanisms. Specifically, the CL500-29 marine group and *Sphaerotilus* contributed to the degradation of protein-like DOM, while the CL500-29 and hgc1 clades played key roles in CRAM formation.

**Discussion:**

The pronounced RDOC enrichment in eutrophic lakes compared to non-eutrophic lakes, rivers, and marine systems underscores the potential of eutrophic lakes to function as significant carbon sinks, highlighting the necessity of integrating bloom-driven RDOC accumulation into carbon budget frameworks to reassess the long-term carbon sequestration potential of these systems.

## 1 Introduction

Inland waters, including lakes, reservoirs, and rivers, represent critical zones for the turnover of organic matter, facilitating the transfer of carbon among terrestrial, marine, and atmospheric systems through biological and photochemical processes. The global carbon footprint of lakes and reservoirs is significant, estimated at 0.73–2.41 Pg CO_2_-eq·yr.^−1^ (Tranvik et al., 2009; Soued et al., 2022). Prior studies have predominantly characterized inland lakes and reservoirs as sources of atmospheric CO_2_, primarily due to increased CO_2_ emissions associated with warming (Sobek et al., 2006; Armstrong, 2010; Tranvik et al., 2018). However, certain inland lakes are currently undergoing intensified eutrophication driven by expanded industrial and agricultural activities, along with the urbanization. As eutrophication progresses, these lakes absorb greater amounts of CO_2_ through algal blooms during summer and autumn, periods marked by extensive algal proliferation (Qi et al., 2023), thereby functioning as net sinks for atmospheric CO_2_.

Concerning the fate of CO_2_ assimilated by algae, many studies proposed that a portion is converted into primary production, which is subsequently transferred to higher trophic levels (e.g., heterotrophic bacteria, zooplankton). This process generates particulate organic carbon that settles and becomes sequestered in sediments, ultimately acting as a carbon sink through a mechanism commonly referred to as the biological carbon pump (Low-Décarie et al., 2014; Meerhoff et al., 2022).

In exploring the carbon sequestration in aquatic systems, Jiao et al. (2010) proposed the microbial carbon pump (MCP) concept, whereby microorganisms assimilate labile dissolved organic carbon (LDOC; lifetime < ~1.5 yr) (Hansell, 2013) and subsequently produce more RDOC. Through these microbial processes, dissolved organic carbon (DOC) pools become increasingly recalcitrant, with RDOC exhibiting lifetimes on the order of decades to millennia (Lønborg et al., 2018). Lechtenfeld et al. (2015) demonstrated that bacterially produced DOM closely resembles naturally occurring marine DOM in terms of chemical composition and structural complexity, underscoring the pivotal role of bacteria in determining the recalcitrance of marine DOM.

Microbially mediated accumulation of RDOC has also been documented in coastal environments using Fourier transform ion cyclotron resonance mass spectrometry (FTICR/MS). Following green tide (*Ulva prolifera*) events, RDOC was conserved during the degradation of macroalgal biomass (Chen et al., 2020; Li et al., 2023). Specifically, (Zhang et al., 2023) reported that 7.8% of carbon in kelp biomass was converted into LDOC, with 0.3% ultimately transformed into RDOC through degradation processes. Consequently, kelp-derived RDOC constitutes a significant component of coastal blue carbon.

However, these concepts and findings have predominantly been established in marine systems, and it remains unclear whether RDOC accumulation similarly occurs in inland lakes, particularly eutrophic lakes characterized by high microbial abundance and metabolic activity.

Recent studies have revealed that colored dissolved organic matter (CDOM) pools in eutrophic lakes exhibit greater recalcitrance compared to those in non-eutrophic lakes, with a higher proportion of CRAM, which serve as indicators of RDOC, detected in eutrophic lake DOM through FTICR/MS analysis (Wen et al., 2022). Several studies have reported the presence of a substantial quantity of CRAM within the DOM of Lake Ontario, a eutrophic lake, as determined by nuclear magnetic resonance (NMR) spectroscopy (Lam et al., 2007). Similarly, CRAM have been detected during algal bloom events in another eutrophic lake, Lake Taihu in China (Zhang et al., 2014). Additionally, recalcitrant proteinaceous material has been shown to retain carbon for over 100 yr. within the water column of high-elevation lakes (Goldberg et al., 2015). However, unlike the deep ocean where RDOC pools persist on centennial to millennial timescales owing to prolonged water residence times (WRT), shallow eutrophic lakes like Taihu are characterized by dynamic hydrological exchange (WRT < 1 year). In these systems, carbon cycling is dominated by high-intensity microbial processing rather than passive storage (Catalán et al., 2016). Recurrent blooms fuel rapid microbial transformation of LDOC, generating reworked DOM fast enough to partially offset hydrological flushing and sustain RDOC enrichment (Zhao et al., 2019).

These observations suggest that RDOC formation may serve as a carbon sink in eutrophic lacustrine environments. Despite its significance, lake RDOC represents an important yet understudied carbon sink, with its characteristics and transformation mechanisms remaining largely unresolved.

Quantification of CRAM by FTICR/MS is constrained by ionization efficiency. In contrast, NMR spectroscopy offers a non-destructive analytical approach requiring relatively simple sample preparation. 1D NMR has been employed to directly elucidate DOM structural features, while 2D NMR enables detailed characterization of the chemical structures of nearly all major organic compounds present in samples (Hertkorn et al., 2006).

It is hypothesized that RDOC is produced by cyanobacterial blooms and subsequently accumulates in eutrophic lakes, thereby representing a significant potential for global carbon sequestration. The primary objectives of this study were: (1) to compare the molecular composition and properties of DOM during bloom and non-bloom periods; (2) to quantify RDOC mediated by bacterial activity; and (3) to estimate the recalcitrant carbon stocks of eutrophic lakes worldwide. Inland lakes are vital to human survival, and the research results aim to elucidate the contribution of eutrophic lakes to carbon sequestration, as well as to support efforts in monitoring the carbon footprints of inland aquatic systems.

## 2 Materials and methods

### 2.1 Sampling and environmental parameters analysis

To elucidate the compositional and structural variations of DOM molecules and the accumulation of RDOC during algal blooms in eutrophic lakes, a seasonal study was conducted in Lake Taihu, situated in the Yangtze River Delta. Lake Taihu is a large (2,338 km^2^), shallow (~1.9 m), eutrophic lake characterized by high microbial abundance and metabolic activity (Wu et al., 2023). Surface water samples were collected monthly, mid-month, from January to December 2014 at a fixed station in Meiliang Bay (31°24′N, 120°13′E, Supplementary Figure S3), using sterile samplers. Meiliang Bay, located in the northern region of Lake Taihu, is a semi-enclosed, hyper-eutrophic bay that historically experiences the most severe and frequent cyanobacterial blooms (*Microcystis* spp.) within the lake (Qin et al., 2010). Meiliang Bay was selected as the study site because it represents the typical bloom-impacted regions of Lake Taihu, which cover a significant portion of Lake Taihu (up to 30–40% during peak season; Shi et al., 2015). It serves as a typical system for investigating the coupling between cyanobacterial proliferation and DOM restructuring, a process that is increasingly relevant as bloom coverage expands in shallow lakes worldwide.

*In situ* measurements of water temperature (*T*) and pH were performed. Samples were subsequently transported to the laboratory in an icebox. Upon arrival, samples were immediately filtered through 0.45 μm pore size nylon filters (32 mm diameter; Rephile Bioscience Ltd., China) and stored at −20 °C until analysis. One liter of surface water was filtered immediately and subjected to solid phase extraction (SPE) using styrene divinyl benzene polymer cartridges (PPL) following the protocol described by Zhang et al. (2014), all SPE-PPL extracts were stored at −20 °C for NMR analysis. The SPE extraction efficiency was approximately 45 ± 4% (Supplementary Table S5).

Nutrient concentrations [NO_3_^−^, NO_2_^−^, NH_4_^+^, dissolved inorganic phosphorus (DIP), and dissolved silicate (DSi)] were determined using an automatic analyzer (Skalar SANplus146). Chlorophyll-a (Chl-a) concentrations were measured according to the method of Pápista et al. (2002). Phytoplankton identification and enumeration were performed using a Sedgwick-Rafter counting chamber under a standard light microscope (OLYMPUS C41) as described by Bi et al. (2010). DOC concentrations were quantified using a total organic carbon analyzer (TOC-VCPN, Japan). Additional physical and hydrological data for the sampling sites are provided in Supplementary Table S1.

### 2.2 FDOM

The parameters employed to obtain the CDOM spectra have been previously documented (McKnight et al., 2001; Guéguen et al., 2014). The principal components of fluorescent dissolved organic matter (FDOM) were analyzed using fluorescence spectroscopy in addition to parallel factor analysis (Stedmon and Bro, 2008).

Excitation-emission matrices (EEMs) were acquired using a Hitachi F-4700 fluorescence spectrophotometer (Japan) equipped with a 1-cm quartz cuvette. Excitation wavelengths ranged from 220 to 450 nm at 5 nm intervals, while emission wavelengths spanned 230 to 600 nm at 2 nm intervals, with a scanning speed of 6,000 nm/min. Raman peaks and Rayleigh scattering were removed from the EEMs. Milli-Q water, analyzed daily, served as a blank, and its spectrum was subtracted from each sample spectrum to correct for the Raman effect.

EEM data analysis was performed using the DOM Fluor toolbox within MATLAB (2019, MathWorks, United States). Detailed parameters and code are available in Stedmon and Bro (2008). The derived fluorescent components were subsequently compared with the OpenFluor database^1^ to determine the composition and fundamental characteristics of each component. Calculation methods for CDOM and FDOM parameters are provided in the Supplementary material.

Briefly, monthly DOM fluorescence was calibrated to the water Raman peak area, with results normalized to Raman units (R.U.) (Stedmon et al., 2011). SUVA_254_ was used to represent the aromatic compound content in DOM, with higher values indicating greater aromaticity. The Humification Index (HIX) characterized the degree of DOM humification (Huguet et al., 2009). The Fluorescence Index (FI) served as an indicator of DOM sources in natural waters, distinguishing between allochthonous and autochthonous origins; low FI values (~1.4) correspond to degraded plant and soil organic matter, whereas high FI values (~1.9) indicate bacterial and algal extracellular release and leachates (McKnight et al., 2001). The Biological Index (BIX) provided an additional measure of DOM source, reflecting the contribution of recently produced DOM from planktonic or microbial sources. Values of 0.6 < BIX < 0.7 suggest a low autochthonous DOM contribution, while BIX > 1 indicates a substantial input of recently produced microbial autochthonous DOM components (Huguet et al., 2009). The Freshness Index (β:α) was defined as the ratio of two fluorescent components, where *β* represents fresher DOM and *α* represents to highly decomposed DOM (Parlanti et al., 2000).

### 2.3 ^1^H NMR spectroscopy

The NMR analyses were conducted following the methods described by Hertkorn et al. (2006) and Zhang et al. (2014). Specifically, 3–5 mg of freeze-dried solid-phase extraction of DOM (SPE-DOM) were dissolved in deuterated methanol and analyzed using a Bruker Avance DRX 500 NMR spectrometer (Bruker, Billerica, MA, United States) equipped with a 5-mm broadband double-resonance probe at 298 K (Dittmar et al., 2008). Solution-state ^1^H NMR spectra were acquired by performing 3,200 scans with an acquisition time of 3 s·scan^−1^. Solvent signal suppression was achieved using the Bruker PRESAT system during spectral acquisition. Baseline correction and spectral integration were performed using MestReNova software (version 14.2.3).

Diffusion-edited (DE) NMR experiments were carried out in accordance with Goldberg et al. (2015), employing a bipolar pulse longitudinal encode-decode sequence. Data acquisition involved 1,024 scans with a 2.5 ms, 333 mT·m^−1^ sinusoidal gradient pulse, a diffusion delay of 50 ms, 16K temporal domain points, and a sample temperature maintained at 298K. The diffusion delay and gradient strength were selected to preferentially attenuate signals from small, highly diffusive molecules while retaining signals from more slowly diffusing macromolecular and/or aggregated DOM components (Chen et al., 1995). This parameter combination has been demonstrated to effectively distinguish between low-molecular-weight, labile compounds and recalcitrant DOM fractions, including CRAM-rich components, in natural aquatic samples (Lam et al., 2007; Goldberg et al., 2015). 1D spectral data were apodized by multiplication with an exponential decay corresponding to 1 Hz line broadening, and zero-filling was applied with a factor of 2.

The absolute concentration of CRAM for each month was calculated as the product of the *in situ* DOC concentration, the carbon recovery efficiency of SPE-PPL extraction, the relative integral abundance of CRAM derived from ^1^H NMR spectra, and a stoichiometric correction factor to adjust for the *H*/*C* ratio discrepancy between CRAM and bulk DOM. Since ^1^H NMR quantifies hydrogen resonance, converting signal intensity to carbon concentration requires an assumption about the *H*/*C* ratio. To account for this, we applied a correction factor derived from FTICR-MS data from Lake Taihu (Zhang et al., 2014), in which the *H*/*C*wa of CRAM-like molecules ranges from 1.22 to 1.26, compared to 1.08 to 1.16 for bulk DOM. This results in a conversion factor ranging from 0.86 to 0.95 (average: 0.90) (see Supplementary material for more details).

### 2.4 DNA extraction, PCR amplification and sequencing data

Total DNA was extracted from frozen filters (0.22 μm pore size polycarbonate filters, Whatman) using the FastDNA^®^ Spin Kit (MP Biomedicals, United States) in accordance with the manufacturer’s protocol. The extracted DNA was evaluated by electrophoresis on a 1% agarose gel, and its concentration and purity were assessed using a NanoDrop 2000 UV-Vis spectrophotometer (Thermo Scientific, Wilmington, United States). The hypervariable V4–V5 region of the bacterial 16S rRNA gene was amplified using the primer pair 515F (5′-GTGCCAGCMGCCGCGGTAA-3′) and 907R (5′-CCGTCAATTCMTTTRAGTTT-3′) on an ABI GeneAmp^®^ 9,700 PCR thermocycler (ABI, CA, United States). The PCR amplification protocol consisted of an initial denaturation at 95 °C for 3 min, followed by 25 cycles of denaturation at 95 °C for 30 s, annealing at 55 °C for 30 s, and extension at 72 °C for 45 s, with a final extension at 72 °C for 10 min, and a hold at 10 °C. The PCR reaction mixture (20 μL total volume) contained 4 μL of 5 × *TransStart* FastPfu buffer, 2 μL of 2.5 mM dNTPs, 0.8 μL of forward primer (5 μM), 0.8 μL of reverse primer (5 μM), 0.4 μL of TransStart FastPfu DNA Polymerase, 10 ng of template DNA, and nuclease-free water to volume. All PCR reactions were performed in triplicate. The PCR products were excised from a 2% agarose gel and purified using the AxyPrep DNA Gel Extraction Kit (Axygen Biosciences, Union City, CA, United States) following the manufacturer’s instructions, and subsequently quantified with a Quantus™ Fluorometer (Promega, United States).

Purified amplicons were pooled in equimolar concentrations and subjected to paired-end sequencing using an Illumina MiSeq PE300 platform (Illumina, San Diego, United States) following the standard protocols provided by Majorbio Bio-Pharm Technology Co. Ltd. (Shanghai, China). The raw sequencing reads have been deposited in the National Center for Biotechnology Information (NCBI) Sequence Read Archive (SRA) under accession numbers SRR23281899 to SRR23281909.

The raw 16S rRNA gene sequencing reads were demultiplexed and quality-filtered using fastp version 0.20.0 (Chen et al., 2018) Subsequently, the reads were merged with FLASH version 1.2.7 (Magoč and Salzberg, 2011) according to the following criteria: (i) 300 bp reads were truncated at any position where the average quality score within a 50 bp sliding window fell below 20; truncated reads shorter than 50 bp and those containing ambiguous nucleotides were discarded; (ii) only overlapping sequences exceeding 10 bp were assembled based on their overlapping regions, with a maximum allowed mismatch ratio of 0.2 in the overlap; reads that could not be assembled were excluded; (iii) samples were differentiated based on barcode and primer sequences, with sequence orientation adjusted accordingly. Barcode matching required exact correspondence, while primer matching allowed up to two nucleotide mismatches.

Operational taxonomic units (OTUs) were clustered at a 97% similarity threshold (Stackebrandt and Goebel, 1994; Edgar, 2013) using UPARSE version 7.1 (Edgar, 2013). Chimeric sequences were subsequently identified and removed. The taxonomy of each representative OTU sequence was assigned using the RDP Classifier version 2.2 (Wang et al., 2007) against the 16S rRNA database (e.g., SILVA v138) with a confidence threshold of 0.7.

### 2.5 Statistical analysis

The data visualization software utilized in this study was Prism version 9.0.0. Additionally, a two-tailed, unpaired Student’s *t*-test was conducted using the same software. Asterisks (“*,” “**,” and “***”) denote statistically significant differences at the 0.05, 0.01, and 0.001 levels, respectively. Hierarchical Cluster Analysis (HCA), Non-Metric Multidimensional Scaling (NMDS), and Redundancy Analysis (RDA) were performed using the free online Majorbio I-Sanger Cloud Platform.^2^

## 3 Results and discussion

### 3.1 Temporal patterns of DOC concentrations and environmental parameters

**Figure 1:**
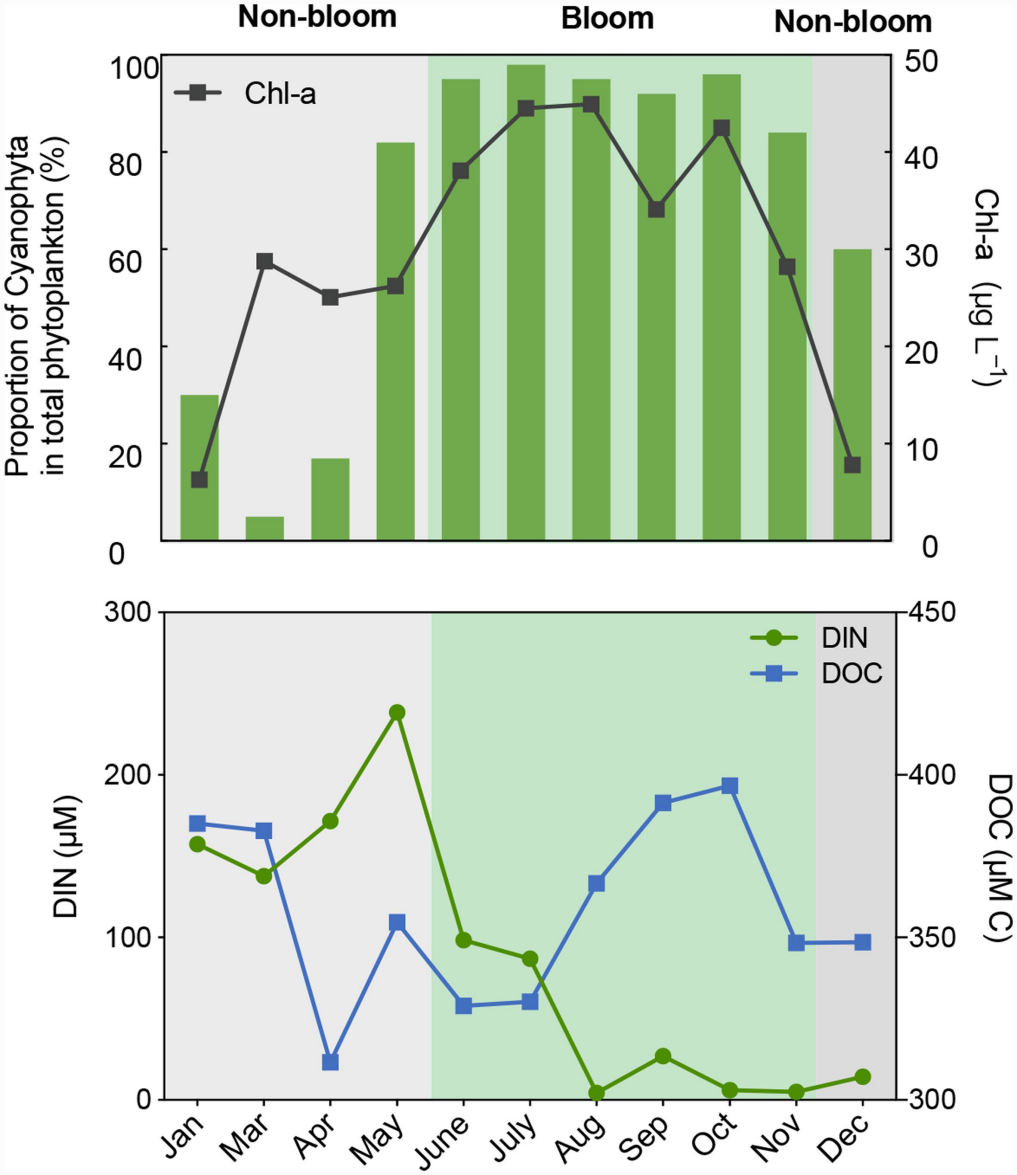
Temporal patterns of DOC concentrations and associated environmental parameters in Lake Taihu.

Bloom and non-bloom periods were defined using an integrated approach that considered chlorophyll-a concentrations, sustained Cyanophyta dominance, official monitoring records (Jiangsu Environment Monitoring Platform)^3^ and persistence over consecutive months. Consequently, June to November was designated as the bloom period, characterized by sustained Cyanophyta dominance and recurrent surface scums (Figure 1 and Supplementary Table S2). The remaining months (January–May and December) were classified as non-bloom. Notably, May was excluded from the bloom period despite elevated phytoplankton biomass (Figure 1), as it lacked the aggregated surface scums and official alerts indicative of fully developed bloom conditions.

The DOC concentrations ranged from 311.57 to 396.69 μM C, with an average value of 358.61 ± 28.29 μM C. No significant differences (*p* > 0.05) in DOC concentrations were observed between the bloom (360.38 ± 29.54 μM C) and non-bloom (356.49 ± 30.00 μM C) periods (Supplementary Table S2). However, DOC concentrations increased following the onset of cyanobacterial blooms and decreased after bloom termination in early October, indicating that cyanobacterial blooms represent a significant source of DOC in Meiliang Bay, Lake Taihu. In contrast to the DOC trends, dissolved inorganic nitrogen (DIN = NH_4_^+^ + NO_3_^−^ + NO_2_^−^) concentrations continued to decline after the cyanobacterial bloom (primarily *Microcystis aeruginosa*), likely due to the high nitrogen demand associated with cyanobacterial growth and reproduction (Li J. et al., 2016), a detailed discussion is provided in Section 3.4.

### 3.2 Optical properties of DOM during bloom vs. non-bloom period

The FDOM plays a crucial role in determining the optical properties and driving biogeochemical processes within aquatic ecosystems. Considering the rising incidence of cyanobacterial blooms in freshwater environments and their capacity to modify the composition of DOM, it is essential to investigate the interactions between these blooms and the characteristics of FDOM.

**Figure 2:**
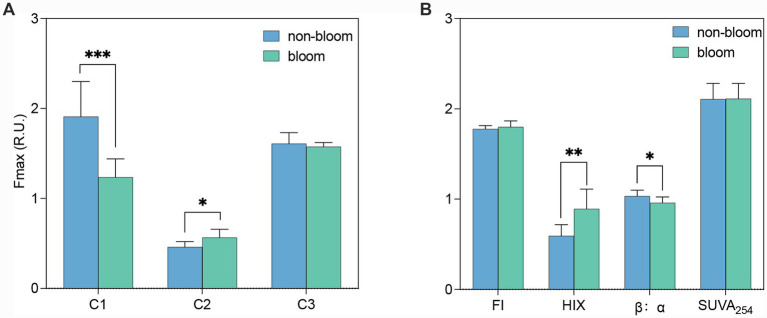
Comparison of FDOM components **(A)** and optical parameters **(B)** between non-bloom period (Jan–May and Dec) and the bloom period (June–Nov). *p < 0.05; **p < 0.01; ***p < 0.001.

The EEMs of DOM exhibit multiple fluorophore peaks that provide insights into the sources, chemical properties, and reactivity of DOM (Coble et al., 1990; McKnight et al., 2001; Fellman et al., 2011). During the sampling periods, three fluorescence components of DOM in Lake Taihu were identified using the PARAFAC method: protein-like C1 and C3, and terrestrial humic-like C2 (Supplementary Figure S3 and Supplementary Table S3). C1 (280 (230)|318 nm) was attributed to autochthonous tyrosine-like fluorescence (Stedmon and Markager, 2005a; Murphy et al., 2008; Yamashita and Tanoue, 2008). C2 (255 (335)|442 nm) corresponded to peaks “A” and “C” characteristic of terrestrial humic substances (Stedmon and Markager, 2005b; Murphy et al., 2008; Yamashita and Tanoue, 2008). C2 (255 (335)|442 nm) corresponded to peaks “A” and “C” characteristic of terrestrial humic substances (Coble, 1996; Yamashita and Tanoue, 2008; Williams et al., 2010). C3 (235|344 nm) resembled tryptophan-like fluorescence and free or bound protein-like components with predominant autochthonous origins, consistent with previously reported spectral features (Murphy et al., 2008; Kowalczuk et al., 2010; Williams et al., 2010). C1 and C3 together accounted for more than 80% of the FDOM. The fluorescence intensity of C1 was significantly lower during the bloom period compared to the non-bloom period (*p* < 0.001), whereas C2 intensity increased during the bloom phase (*p* < 0.05). No significant change was observed in C3 fluorescence intensity between the bloom and non-bloom periods (Figure 2A).

The FI was employed to distinguish between aquatic microbial and terrestrial sources of DOM (McKnight et al., 2001). In Meiliang Bay, the FI of DOM was approximately 1.8 (1.79 ± 0.03) (Figure 2B), with no significant difference observed between cyanobacterial bloom and non-bloom periods. This result suggests that FDOM was predominantly derived from microbial sources year-round, consistent with observations in other lakes (McKnight et al., 2001).

During the bloom period, the HIX was significantly elevated (*p* < 0.001) compared to the non-bloom period (Figure 2B), indicating a relatively higher degree of DOM humification and decomposition during the bloom season. However, HIX values remained relatively low during both periods (ranging from 0.46 to 1.27, with a mean of 0.77 ± 0.25) (Supplementary Table S4), reflecting a generally weak degree of humification. Similarly, HIX values in the overlying water of Taihu Lake ranged from 0.26 to 1.94, further indicating a low level of humification (Wang et al., 2020). Furthermore, the HIX values in Lake Taihu were significantly lower than those reported for Lake Dianchi (4.25) (Du et al., 2016), Lake Baihua (2.88–5.63) (Song et al., 2019), Lake Changshou (3.10–6.47) (Jiang et al., 2018), and Lake Michigan (2.33) (DeVilbiss et al., 2016), suggesting weaker humification of DOM in Lake Taihu. The relatively low humification degree (low HIX) in Lake Taihu reflects the combined influence of DOM source characteristics and subsequent microbial processing (Figure 2B). Unlike lakes dominated by terrestrial humic substances (e.g., Lakes Dianchi, Michigan, Baihua, and Changshou; DeVilbiss et al., 2016; Du et al., 2016; Jiang et al., 2018; Song et al., 2019), Taihu receives massive inputs of fresh, protein-like DOM from cyanobacterial blooms, which inherently exhibit low aromaticity and limited humification (Zhang et al., 2009; Zhang et al., 2010; Wen et al., 2022). Furthermore, rapid microbial turnover of these labile substrates constrains the accumulation of highly humified compounds (Zhou et al., 2021; Liu et al., 2022). Consequently, while bloom-associated microbial activity induces a relative increase in HIX, absolute values remain low (Figure 2B), indicating that processing enhances humification signals but acts predominantly on fresh substrates.

Consistent with the findings of HIX, the β:α ratio was significantly lower during the bloom season compared to the non-bloom season (*p* < 0.05) (Figure 2B), indicating a higher proportion of fresh DOM in the non-bloom period and a greater degree of DOM decomposition during the cyanobacterial bloom. SUVA_254_, which represents the aromaticity of DOM (Weishaar et al., 2003). SUVA_254_ values did not show significant differences between the bloom and non-bloom groups, both being approximately 2.0 L·mg^−1^·m^−1^ (Figure 2B and Supplementary Table S4), suggesting that CDOM was not highly aromatized. These values are lower than those in eutrophic Lakes Gaoyou (3.69) and Dongping (3.51) (Wen et al., 2022). Falling between typical terrestrial (2.5 to >4.0 L·mg^−1^·m^−1^) and marine (<1.5–2.0 L·mg^−1^·m^−1^) ranges (Weishaar et al., 2003; Helms et al., 2008; Jaffé et al., 2008). Lake Taihu’s intermediate value (~2.0 L·mg^−1^·m^−1^) reflects a DOM pool dominated by autochthonous or processed components rather than fresh terrestrial humics. This relatively low aromaticity results from multiple interacting mechanisms: photochemical degradation facilitated by significant UV penetration in the shallow (~1.9 m) water column (Fleischmann, 1989); dilution of terrestrial aromatic signals by massive aliphatic, protein-like DOM during cyanobacterial blooms (Zhang et al., 2009); oxidative dearomatization converting aromatic precursors (e.g., polyphenols) into non-aromatic structures under photo- or bio-oxidative conditions (Li et al., 2024); and continuous microbial reshaping of terrestrial DOM along the aquatic continuum (Guo et al., 2025).

The FDOM is commonly used as a proxy for DOC in aquatic systems (Li et al., 2022). The proteinaceous fraction of FDOM is generally regarded as an indicator of LDOC (Yang et al., 2015). The production of protein-like C1 and C3 is believed to result from the microbial degradation of algal cells (Stedmon and Markager, 2005b; Zhang et al., 2009). The fluorescence intensity of these protein-like C1 and C3 was significantly higher than the terrestrial humic-like C2 (*p* < 0.0001) (Figure 2A), suggesting that a substantial proportion of LDOC may originate from cyanobacterial blooms rather than terrestrial sources. Cyanobacterial cells contain large amounts of protein-like substances, which are released into the surrounding water during extracellular release and/or following cell apoptosis or death (Lee et al., 2018). However, the concentration of protein-like components C1 during bloom periods was significantly lower than during non-bloom periods (Figure 2A). This observation suggests that protein-like substances are rapidly consumed by heterotrophic bacteria during cyanobacterial blooms, as further supported by evidence indicating that FDOM is less fresh and more humified during bloom periods (Figure 2B).

### 3.3 Changes of DOM composition as determined based on NMR

**Figure 3:**
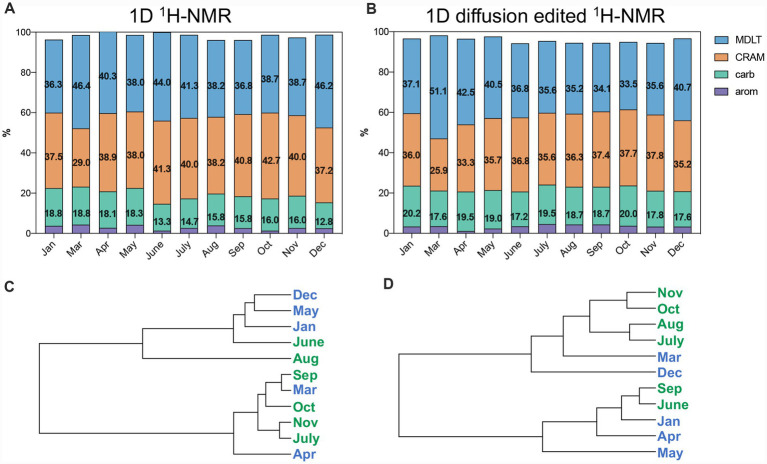
^1^H NMR **(A)** and DE-^1^H NMR **(B)** section integrals for key substructures of TH-DOM; hierarchical cluster dendrogram based on ^1^H NMR **(C)** and DE-^1^H NMR **(D)** section integrals (0.01 ppm resolution) (note: carb, carbohydrates; arom, aromatic and phenolic constituents; MDLT, material derived from linear terpenoids; CRAM, carboxyl-rich alicyclic molecules. The spectra were normalized to the identical total NMR integral).

In this study, 1D proton NMR (1D ^1^H NMR) and DE-^1^H NMR techniques were employed to provide a comprehensive and detailed characterization of the molecular composition and temporal dynamics of DOM in Lake Taihu (TH-DOM). Additionally, the study rigorously investigated the potential impacts of cyanobacterial blooms on the compositional profile of TH-DOM. Quantification of the relative abundances of four DOM component groups—aromatic and phenolic constituents (arom), carbohydrate (carb), CRAM, and material derived from linear terpenoids (MDLT)—was achieved using 1D ^1^H NMR spectroscopy (Figures 3A,B).

The ^1^H NMR spectra obtained from TH-DOM (Supplementary Figure S4) closely resembled those reported for DOM from various marine and freshwater environments in the literature (Hertkorn et al., 2006, 2013; Lam et al., 2007). The combined area integrals of these four spectral regions accounted for approximately 96% of the TH-DOM. Terpenoids, a subclass of prenyl lipids produced by *Cyanobacteria*, may constitute the predominant structural components of TH-DOM (~73%), including CRAM and MDLT fractions (Figure 3A). A similar DOM composition has been observed in other eutrophic freshwater lakes, such as Lake Ontario (Lam et al., 2007).

**Figure 4:**
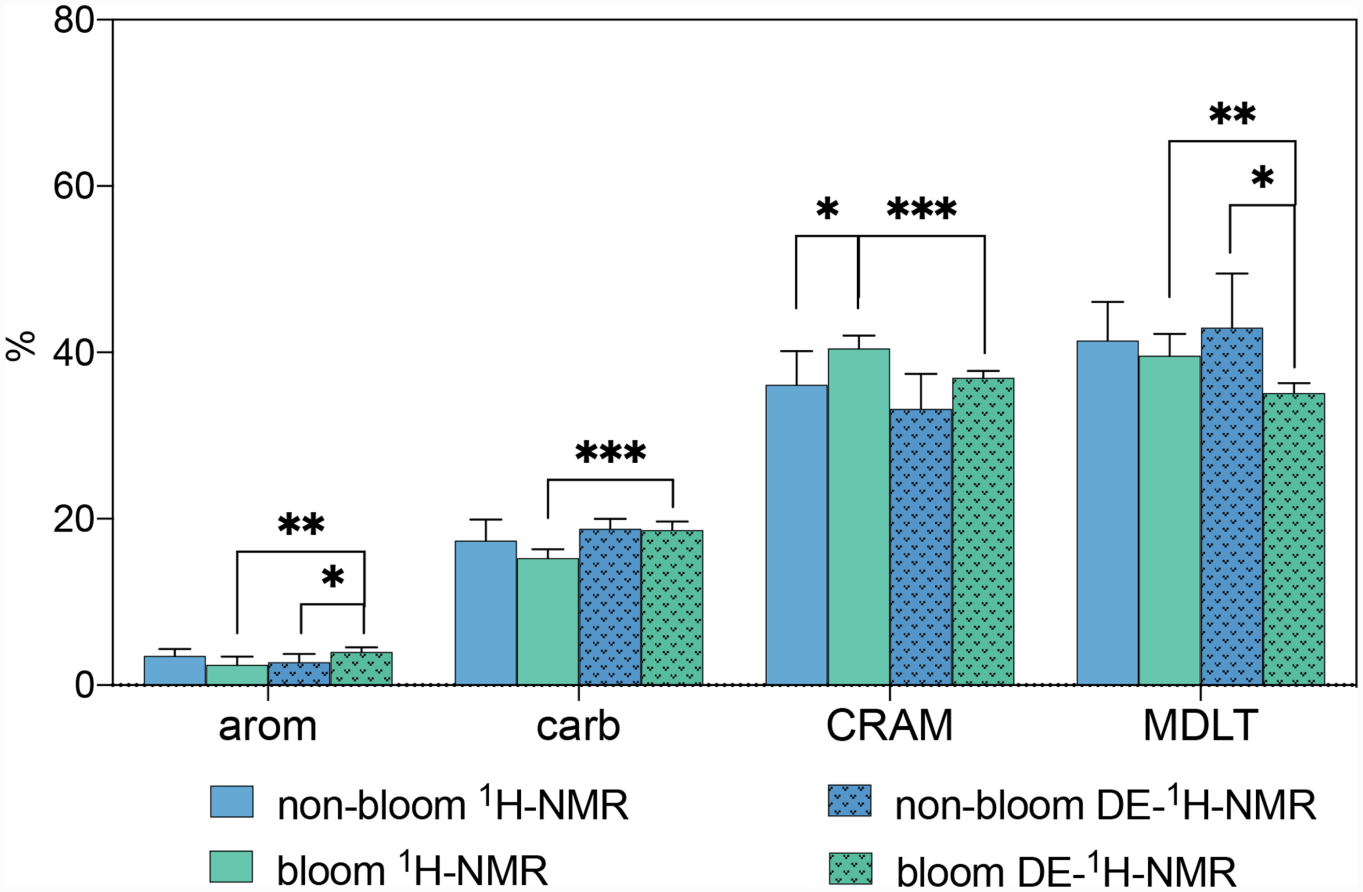
Major molecular components of DOM during non-bloom and bloom periods based on 1D ^1^H NMR and DE-^1^H NMR (carb, carbohydrates; arom, aromatic and phenolic constituents; MDLT, material derived from linear terpenoids; CRAM, carboxyl-rich alicyclic molecules).

Aromatic and phenolic constituents did not exhibit significant differences between the bloom and non-bloom periods (Figure 4). These compounds represented less than 5% of the ^1^H resonances across all samples (Figures 3A,B), a value lower than the 8% reported for DOM in Lake Ontario (Lam et al., 2007), but higher than the less than 1.5% observed in Pacific surface seawater (Hertkorn et al., 2006).

Carbohydrates comprised 11–19% of the TH-DOM (Figure 3A), which is lower than the levels recorded in 2007 in Lake Taihu (25.5% in June; 19.0% in November) and comparable to the 17% reported for Lake Ontario (Lam et al., 2007; Zhang et al., 2014). These proportions in both freshwater lakes were lower than those reported for the Pacific Ocean (23–33%) (Hertkorn et al., 2006). Furthermore, the carbohydrate content did not differ significantly between bloom and non-bloom periods (Figure 4).

The proportion of MDLT ranged from 36 to 47% (Figure 3A), consistent with data from 2007 (39.4% in June; 44.6% in November) and higher than unpublished data from 2009 to 2010 (27–39%). No significant difference in MDLT proportion was observed between bloom and non-bloom periods.

The CRAM accounted for 29–43% of the composition, which aligns with results from 2009–2010 (27–36%, unpublished data) (Figure 3A). These proportions were lower than those observed in Lake Ontario DOM (62%), higher than in Pacific surface seawater (17–28%), and comparable to Pacific bottom seawater (approximately 37%) (Hertkorn et al., 2006; Lam et al., 2007). Notably, the proportion of CRAM increased significantly during the bloom period compared to the non-bloom period (*p* < 0.05) (Figure 4).

The bioavailability of DOC is primarily affected by its molecular composition within a given environment (Kellerman et al., 2015). CRAMs are regarded as a form of RDOC present in both marine and freshwater systems (Arakawa and Aluwihare, 2015; He et al., 2022). Although the precise chemical structure of CRAMs remains unresolved due to their complexity and heterogeneity, they are generally characterized by alicyclic structures—cyclic carbon compounds that are non-aromatic—and a high abundance of carboxyl functional groups. The alicyclic framework and carboxyl group richness confer properties such as water solubility, acidity, and metal complexation capacity, which collectively contribute to their resistance to biodegradation and recalcitrance (Hertkorn et al., 2006). These attributes render CRAMs significant in various environmental and geochemical processes (Cai and Jiao, 2023). The significant increase in CRAM (*p* < 0.05) during the bloom period indicates that the cyanobacterial bloom outbreak may trigger the accumulation of RDOC. This phenomenon may be associated with changes in the bacterial community (Section 3.5).

Furthermore, it has been demonstrated that extracellular extracts of cyanobacteria do not serve as a direct source of CRAM (Zhang et al., 2014). Although these extracellular extracts contain a greater variety of labile compounds, upon release into the aquatic environment, these compounds become increasingly recalcitrant due to photochemical processes (Johannsson et al., 2021). Similarly, certain compounds become progressively more recalcitrant through microbial metabolism. This phenomenon is similar to observations in coastal green tides (or macroalgal blooms), which have been identified as contributors to the formation of RDOC (Li et al., 2023). Consequently, the role of heterotrophic bacteria warrants further consideration.

The DE was employed to enhance signals originating from macromolecules and/or aggregated species, as species exhibiting high diffusivity or mobility are not refocused and are effectively suppressed in the resulting spectrum (Chen et al., 1995; Lam et al., 2007; Goldberg et al., 2015). The DE spectra showed profiles comparable to those observed in ^1^H NMR spectra (Supplementary Figure S4), indicating that macromolecular and/or aggregated structures are prevalent in TH-DOM.

Compared to conventional ^1^H NMR spectra obtained during the bloom period, the relative proportions of carbohydrates, aromatic, and phenolic constituents increased following the suppression of small molecule signals, whereas the proportions of CRAM and MDLT decreased (Figure 4 and Supplementary Table S5). Carbohydrates, aromatic, and phenolic constituents during the bloom period predominantly comprised larger molecules. In contrast, CRAM and MDLT exhibited a heterogeneous composition, with contributions from both larger and smaller molecules to their overall signals. These results indicate that carbohydrates, aromatic, and phenolic constituents originate from macromolecular sources, whereas CRAM and MDLT derive from mid- and/or small-sized molecules (Woods et al., 2010). This observation aligns with previous results reported in other freshwater systems (Lam and Simpson, 2008; Wang et al., 2018). Only a minor fraction of carbohydrates in natural waters exists as monomers or simple forms; the majority are present as polymers (e.g., *N*-acetyl amino polysaccharides) in marine environments (Aluwihare et al., 2005), which may explain the increased carbohydrate proportion observed in the DE-^1^H NMR spectrum. CRAM plays a significant role in the aggregation of SPE-DOM, with MDLT contributing to a lesser extent (Lam and Simpson, 2009). The molecular weight of CRAM has been reported to range approximately from 400 to 700 Da (Hertkorn et al., 2006, 2013). The observed reduction in CRAM proportion after suppression of small molecule signals may result from the removal of unaggregated CRAM signals. However, due to the high concentration of the samples, it remains challenging to ascertain whether these species are inherently macromolecular or merely aggregated/associated forms.

The HCA of DE-^1^H NMR data grouped more adjacent months into single clusters compared to ^1^H NMR (Figures 3C,D), suggesting that the macromolecular and/or aggregate components exhibited greater structural similarity across consecutive months. This observation may be attributed to the reduced lability of these macromolecular and/or aggregate components.

### 3.4 Succession of bacterial community composition during cyanobacterial bloom

Understanding the interactions between cyanobacterial blooms and their associated bacterial communities is essential for elucidating the subsequent formation of RDOC during bloom events. To assess bacterial diversity, high-throughput sequencing of the 16S rRNA gene targeting the V4–V5 regions was performed. A total of 639,449 16S rRNA gene sequences were obtained from all samples. Following random resampling, 837 OTUs with a similarity threshold of ≥97% were identified for community analysis. These OTUs were taxonomically classified into 25 phyla, 51 classes, 141 orders, 224 families, 376 genera, and 528 species.

**Figure 5:**
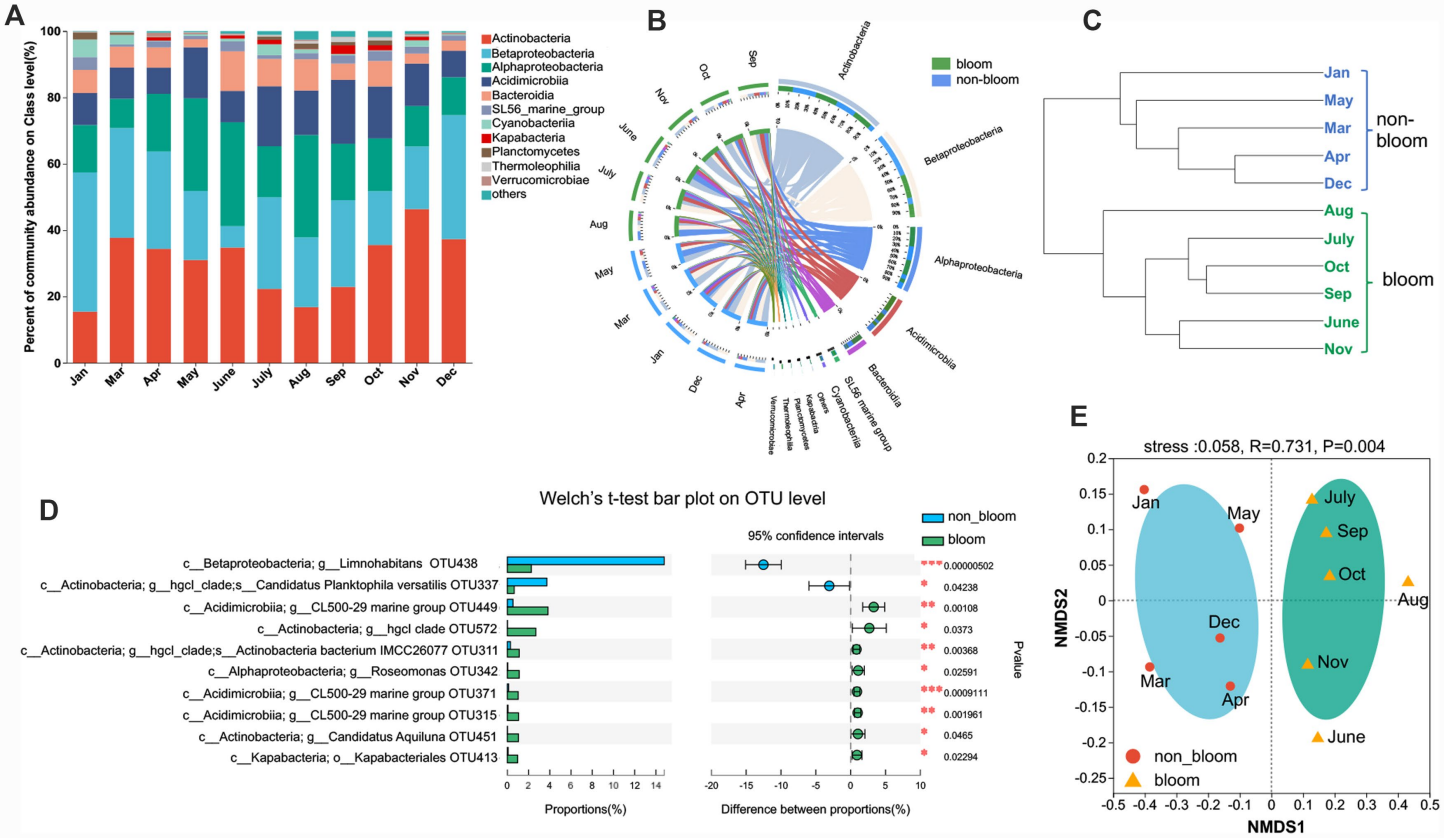
Variations in bacterial communities at class level during the cyanobacterial bloom and non-bloom periods **(A)** and distribution of bacterial community for each sample at class level **(B)**. Extended error bar plot of 10 most abundant OTUs that differ significantly between the non-bloom period and the bloom period **(D)**. HCA **(C)** and NMDS plots **(E)** of bacterial communities across sampling months. The width of the bars from each class indicates the relative abundance of that class in the sample. Others: Sum of taxa with a relative abundance below 1%; Positive differences in mean relative abundance indicate OTUs overrepresented on the bloom group, while negative differences indicate greater abundance in the non-bloom group.

During both non-bloom and bloom periods, the dominant bacterial classes included *Actinobacteria*, *Betaproteobacteria*, *Alphaproteobacteria*, *Acidimicrobiia*, and *Bacteroidia* (Figures 5A,B), which are commonly reported taxa in cyanobacterial bloom environments (Chun et al., 2019; Huang et al., 2022; Lefler et al., 2023). Welch’s *t*-test identified nine classes exhibiting significant differences in abundance between the non-bloom and bloom periods (Supplementary Figure S5). The onset of cyanobacterial bloom significantly altered the dominant bacterial community composition relative to the non-bloom period. Specifically, the relative abundance of *Betaproteobacteria* was significantly higher during the non-bloom period, whereas *Acidimicrobiia* was significantly more abundant during the bloom period (*p* < 0.05). At the OTU level, 51% of OTUs were shared between the non-bloom and bloom groups (Supplementary Figure S6). However, Welch’s *t*-test revealed that OTU438 (genus *Limnohabitans*, 12.6–17.9%) and OTU337 (species *Candidatus Planktophila versatilis*, 0.5–5.8%) were significantly more abundant during the non-bloom period, while OTU449 (genus *CL500-29 marine group*, 1.8–5.4%) and OTU572 (genus *hgc1 clade*, 0.1–6.1%) exhibited significantly higher abundance during the bloom period (Figure 5D). These observed shifts in bacterial community composition between bloom and non-bloom periods may affect the modification of DOM composition.

To elucidate the monthly dynamics of bacterial communities, HCA and NMDS were employed. Both HCA and NMDS results demonstrated a clear division of bacterial communities sampled across different months into two groups (Figures 5C,E). Notably, these two clusters corresponded precisely to the presence or absence of cyanobacterial blooms. The bacterial community profiles within these clusters differed significantly at the OTU level (Bray–Curtis ANOSIM = 0.73, *p* < 0.01), indicating a pronounced succession in bacterial community composition associated with cyanobacterial bloom events. Overall, bacterial community composition exhibited substantial variations throughout the year, driven by cyanobacterial blooms and reflecting important successional patterns.

The ecological functions of bacterial communities were characterized using FAPROTAX analysis, through which 44 microbial functional groups were identified (Yang et al., 2022). For simplicity, representative functional groups were selected to elucidate ecological and metabolic variations.

Given that cyanobacterial carbon fixation requires a higher nitrogen demand than phytoplankton (C:N ratio = ~4 under both iron-limited and iron-replete conditions; Cunningham and John, 2017), cyanobacterial blooms are known to stimulate bacterial communities involved in carbon and nitrogen transformations within lacustrine environments (Li et al., 2018; Yang et al., 2021). Carbon cycle functions encompassed chemoheterotrophy, aerobic chemoheterotrophy, ureolysis, ligninolysis, fermentation, and aromatic compound degradation (Supplementary Figure S7). Nitrogen cycle functions predominantly included nitrate reduction, nitrate respiration, nitrogen respiration (denitrification), and nitrogen fixation. These functions are likely linked to the formation and decomposition of *Cyanobacteria*. Comparable findings were reported by (Chun et al., 2019) in their investigation of cyanobacterial blooms in riverine systems, where aerobic heterotrophic microbes were proposed to be the principal agents of organic matter biodegradation during such bloom events. Metagenomic and metaproteomic analyses showed that carbon transfer from cyanobacteria to heterotrophic populations was efficient (Ataeian et al., 2022), emphasizing their importance in maintaining ecosystem stability during cyanobacterial blooms.

### 3.5 Bacteria-mediated accumulation of RDOC

The succession of bacterial communities represents not merely a taxonomic shift, but a fundamental transformation of the ecosystem’s metabolic potential. The community shifted from *Betaproteobacteria*-dominance (e.g., *Limnohabitans*) during the non-bloom period to an assemblage dominated by *Acidimicrobiia* and *Actinobacteria* (e.g., *CL500-29 marine group* and *hgc1 clade*) during the bloom (Figure 5). This compositional turnover corresponds to a pronounced shift in carbon processing strategies.

**Figure 6:**
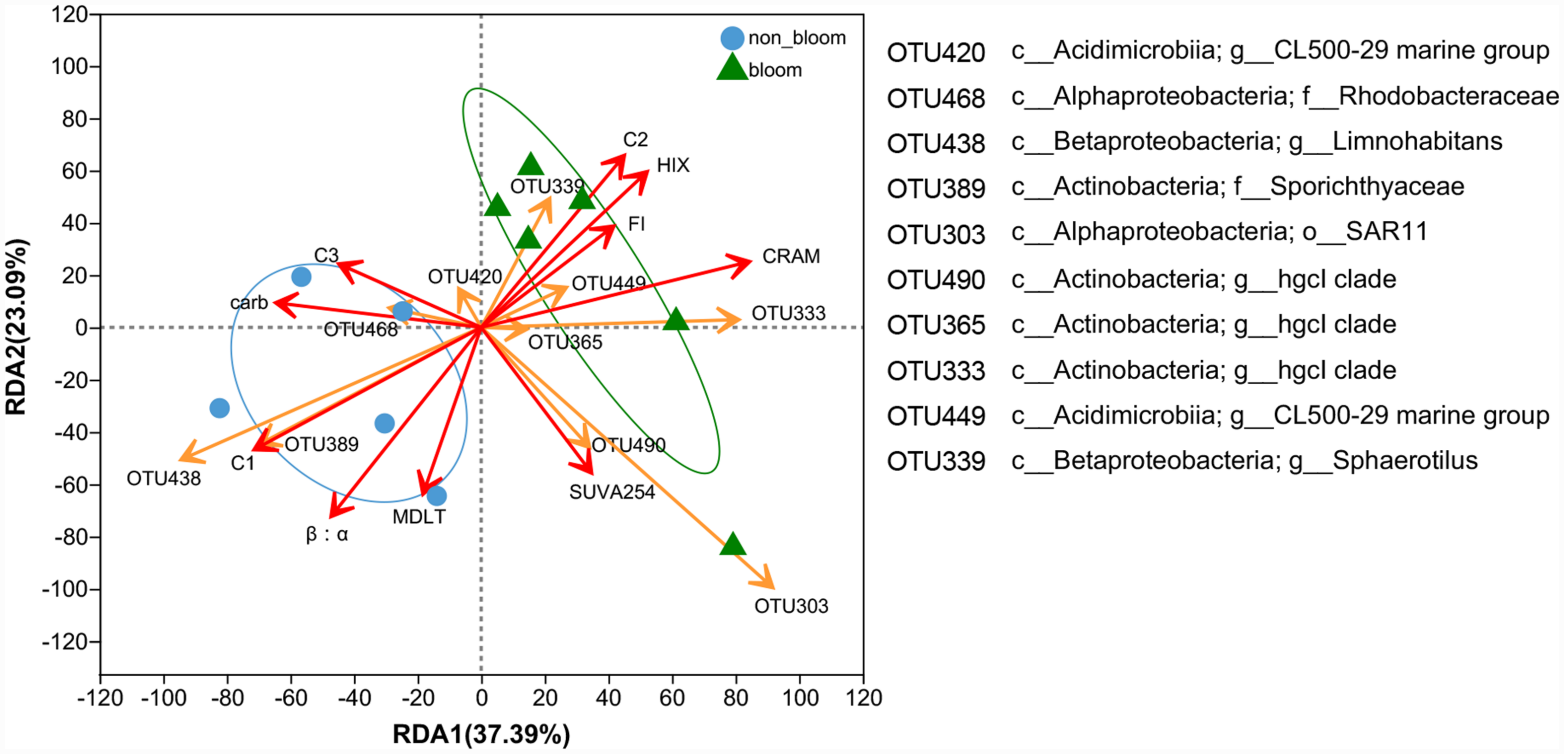
RDA for relationships between specific bacterial taxa and DOM.

During the non-bloom period, *Limnohabitans* (OTU438, *Betaproteobacteria*) was strongly associated with labile protein-like components (C1) (Figure 6), aligning with its established ecological role as an opportunistic heterotroph specialized in the rapid assimilation of low-molecular-weight, labile substrates (Salcher et al., 2013). However, as blooms progressed and labile substrates were progressively depleted, transformed, or sequestered into more complex molecular forms, these taxa declined (Teeling et al., 2012). In contrast, bloom-associated taxa, specifically *CL500-29 marine group* (OTU449) and *hgc1 clade* (OTU572), showed significant positive correlations with CRAM abundance and HIX values (Figure 6), indicating their close involvement in the accumulation of RDOC. Genomic evidence suggests that these taxa possess broad metabolic versatility and the capacity to utilize complex algal-derived substrates, enabling persistence under carbon-limited conditions (Ghylin et al., 2014; Neuenschwander et al., 2018). Similar associations between filamentous bacteria (e.g., *Sphaerotilus*, OTU339) and high organic-matter environments further support this interpretation (Zhang et al., 2020).

Mechanistically, laboratory incubations confirm that microbial reprocessing of algal-derived DOM leads to the rapid generation of increasingly recalcitrant forms (Zheng et al., 2019; Chen et al., 2020; Li et al., 2022), specifically by restructuring alicyclic precursors (e.g., steroids) into CRAM-like molecules through oxidation, ring cleavage, and molecular recombination (Hach et al., 2020; Liu et al., 2023). This mechanism is particularly relevant in eutrophic lakes, where cyanobacteria are known to synthesize abundant alicyclic lipids, including hopanoids (Talbot et al., 2008; Sáenz et al., 2012; Zarzycki et al., 2017), which can serve as effective CRAM precursors (Liu et al., 2023). Although multiple metabolic pathways, including *β*-oxidation, aromatic compound degradation, leucine degradation, and the mevalonate pathway have been implicated in CRAM formation (He et al., 2022), in Lake Taihu, these processes are predominantly mediated by bloom-enriched *Acidimicrobiia* and *Actinobacteria* (Figures 5A,B), consistent with selective microbial enrichment of taxa capable of degrading complex organic matter on cyanobacterial aggregates (Gao et al., 2023).

Predicted functional shifts further support for this bacteria-mediated pathway. Increases in fermentation and hydrocarbon degradation align with enhanced bacterial decomposition of cyanobacterial cells and their derivatives (Supplementary Figure S7). Specifically, hydrocarbons (e.g., C15 and C17 alkanes) synthesized and released by *Microcystis* (Lea-Smith et al., 2015) are degraded by bloom-associated taxa like *CL500-29 marine group* and the *hgc1 clade* (Shen et al., 2022). In late bloom stages, enhanced nitrate reduction and nitrate respiration likely contributed to the rapid decline in nitrate concentrations (Figure 1 and Supplementary Table S2), reflecting high nitrogen demand of processing biomass (Chen et al., 2021; Kokociński et al., 2021). Concurrently, enhanced urease activity during blooms indicated a reliance on organic nitrogen (e.g., urea) to sustain cyanobacterial growth (Moore et al., 2002; Li et al., 2016).

Comparative analysis of *Synechococcus*-based marine microcosms and *Microcystis*-dominated lacustrine blooms reveals that while the MCP universally drives DOM toward similar molecular assemblages, the underlying pathways and temporal regimes differ.

First, a stark contrast exists regarding the visibility of algal signatures. Unlike marine picocyanobacteria (e.g., *Synechococcus*) that can directly release RDOC-like compounds (Zhao et al., 2024), Lake Taihu’s bulk DOM lacks direct signatures of cyanobacterial metabolites (e.g., *Microcystis*) (Zhang et al., 2014). This implies that, in eutrophic lakes, the contribution of cyanobacterial blooms to RDOC is predominantly indirect, mediated through multi-step microbial transformation rather than the preservation of algal metabolites themselves (Zhang et al., 2014). This divergence is likely driven by fundamental metabolic differences between the dominant producer taxa (*Microcystis* vs. *Synechococcus*) and their distinct associated microbial consortia, highlighting that cyanobacterial taxa with streamlined metabolisms and distinct biosynthetic capacities may contribute differently to MCP pathways across systems (Zhang et al., 2014; Zhao et al., 2024).

Second, timescales modulate MCP operation. The marine microcosm emphasizes gradual RDOC accumulation under quasi-steady conditions (Zhao et al., 2024). By contrast, viewed as a multi-year continuum rather than isolated events, the repeated bloom cycles in Lake Taihu facilitate the gradual accretion of microbial transformation residues, effectively functioning as a “pulsed” MCP. Although individual blooms are transient (contrasting with the 720 day co-culture experiments; Zhao et al., 2024) and punctuated by physical disturbance, the decadal recurrence creates a sustained selection environment for RDOC accumulation (Zhang et al., 2014; Xue et al., 2024; Chen et al., 2025). Crucially, these regimes are not mutually exclusive. The low HIX values (Figure 2B) imply a dilution by fresh precursors but should not be conflated with low recalcitrance, as the accumulation of RDOC (e.g., CRAM) can proceed via pathways distinct from optical humification.

Despite these contrasts, both systems document a directional shift toward increased molecular diversity, enhanced carboxylation, and the emergence of CRAM-like structures during sustained microbial processing (Zhang et al., 2014; Zhao et al., 2024). This molecular convergence indicates that CRAM formation represents the universal thermodynamic endpoint of the MCP, independent of whether the precursor originates from a marine autotroph or lacustrine bloom byproducts. It highlights that the MCP is not a single process but a conceptual framework whose manifestation depends critically on ecosystem context. This refined understanding underscores the need to evaluate carbon sequestration potential within an explicitly time-resolved and ecosystem-specific framework.

A limitation of this study is that surface-floating cyanobacterial aggregates were removed during sampling, potentially excluding particle-attached bacterial communities. These attached microbes have been shown to degrade complex biopolymers, including cell wall components (Macdonald et al., 2024), and thus may contribute substantially to carbon transformation during blooms. Therefore, the CRAM increases observed in this study may represent conservative estimates.

### 3.6 Potential contribution of bloom-derived RDOC to carbon sequestration in eutrophic lakes

Eutrophic lakes experiencing recurrent cyanobacterial blooms have increasingly been recognized as active sites of organic carbon transformation, with cyanobacteria-derived DOM potentially contributing to downstream carbon sequestration through microbial reworking and hydrological export.

To provide a first-order estimate of the RDOC pool, CRAM concentrations were estimated based on NMR integrals and H/C conversion factors (see Method sub-section). During the non-bloom period, the estimated CRAM concentration ranged from 49.55 to 54.74 μM C (central estimate: 51.86 ± 11.22 μM C), accounting for the uncertainty in H/C conversion. During the bloom period, the estimated CRAM concentration ranged from 58.10 to 64.18 μM C (central estimate: 60.80 ± 8.21 μM C). This corresponds to an approximate 17% increase in CRAM concentration during the bloom conditions compared to non-bloom conditions within the same year (2014), reflecting a distinct seasonal contrast. Notably, CRAM enrichment occurred without a net increase in total DOC (*p* > 0.05) (Figure 2 and Supplementary Table S2), suggesting a dynamic turnover where microbial conversion of labile algal substrates into recalcitrant CRAM was balanced by simultaneous mineralization or export. This compositional shift underscores active MCP processes in eutrophic systems (Ogawa et al., 2001; Jiao et al., 2010).

Similar RDOC accumulation has also been documented in various algal degradation experiments. For instance, approximately 1.6% of the green-tide macroalgal carbon remained after more than 1.4 yr. (Chen et al., 2020), a value exceeding the estimated RDOC production rates of 0.63% of net community production derived from global DOC distribution patterns (Hansell and Carlson, 1998; Hansell, 2013). In a kelp degradation experiment, the retained RDOC accounted for 58% of DOC, with 15% of RDOC newly produced (Li et al., 2022). Conversely, a 3 year seawater incubation reported a biogenic RDOC production rate below 0.4% (Osterholz et al., 2015). These discrepancies likely arise from inherent limitations in measurement and calculation methods, along with factors such as substrate heterogeneity, microbial diversity, and complex interactions among these components, which may affect microbial carbon processing efficiency. Collectively, these findings support the notion that eutrophic waters where labile substrates and microbial activity are elevated, tend to exhibit higher RDOC accumulation efficiency than non-eutrophic systems.

The annual CRAM average concentration was 56.74 ± 10.29 μM C, representing ~16% of the total DOC. This fraction is ~8% higher than the CRAM-like carbon proportion reported for the Pacific Ocean (Hertkorn et al., 2006). By integrating satellite-derived bloom extent (Shi et al., 2015) with hydrological parameters (average water depth and retention time in Supplementary Table S1), RDOC export from Lake Taihu was estimated at 3.24 ± 0.58 × 10^3^ t C·yr.^−1^_._ Because SPE-PPL recovery varies among water types and PPL columns exhibit a bias toward nitrogen-containing and hydrophobic aliphatic compounds (Dittmar et al., 2008; Perminova et al., 2014; Zherebker et al., 2016), these values should be considered conservative, lower-bound estimates. Despite these limitations, SPE-PPL remains the most widely adopted method for isolating DOM for molecular-level analyses, balancing recovery rate and sample representativeness among currently available extraction techniques (Li et al., 2016; Li et al., 2017).

The extent to which CRAM-rich DOM contributes to carbon sequestration ultimately depends on its persistence. Recent insights challenge the traditional view of CRAM as purely recalcitrant pool, as surface water CRAM can contain a biologically labile fraction of up to 43% (McKee et al., 2024). In Lake Taihu, this implies a dynamic equilibrium where bloom-driven microbial activity and photochemical processes continuously generate CRAM, while heterotrophic microbes selectively consume the more labile fractions. Consequently, the accumulated CRAM represents the metabolic residues of this extensive processing.

Regarding formation, terrestrial precursors (e.g., lignin, polyphenols) complement the primary algal-derived MCP pathway, being reshaped into CRAM via phototransformation (Niu et al., 2019) and oxidative dearomatization (Li et al., 2024). Despite these diverse autochthonous and allochthonous origins as well as the distinct biological and photochemical formation pathways, the environmental fate of these molecules is ultimately convergent. Precursors transformed into specific bicyclic carboxylate-rich alicyclic motifs reach a structural state of thermodynamic stability (Craig et al., 2024), a pathway consistent with recent findings that diverse phytoplankton universally yield recalcitrant CRAM residues (>10%) in marine environment (Lu et al., 2025). This pool is further stabilized via a polarity-dependent mechanism where microbes selectively preserve high-polarity, oxidized variants (Cai et al., 2025). Thus, in both systems, DOM is universally filtered toward this stable bicyclic endpoint regardless of precursor origin (Craig et al., 2024).

Converging evidence across freshwater, coastal, and marine systems indicates that a substantial fraction of microbially processed RDOC is resistant to biological and photochemical decay over timescales sufficient for hydrological export. Short- to medium-term degradation experiments have shown that 45–85% of DOC in stratified bottom and pore waters exhibits no measurable decay over 126 days (Lengier et al., 2024) and approximately 42–46% of seagrass- and macroalgal-derived DOC persists after 1 year of continuous oxygenation, mixing and light exposure (Yamuza-Magdaleno et al., 2024). At broader spatial scales, global analyses of DOC reactivity indicate that this recalcitrant pool displays turnover times on the order of ~450–760 days along the freshwater–estuarine–coastal continuum, with RDOC becoming selectively enriched offshore as more reactive fractions are progressively depleted (LaBrie et al., 2020). These cross-system patterns suggest that CRAM-rich RDOC produced during cyanobacterial blooms in eutrophic lakes is likewise capable of surviving subsequent transport and forming a semi-stable carbon pool, thereby contributing to long-term carbon storage and potentially exerting atmospheric cooling effects (Jiao et al., 2024). Notably, CRAM-like structures do not imply absolute inertness. Certain CRAM components remain susceptible to biological and photochemical degradation under highly productive Arctic conditions (McKee et al., 2024), and simulated ocean warming has been shown to destabilize accumulated RDOC reservoirs by accelerating the microbial turnover of CRAM-like fractions (Zhao et al., 2024), indicating that the stability of CRAM may be context-dependent.

**Figure 7:**
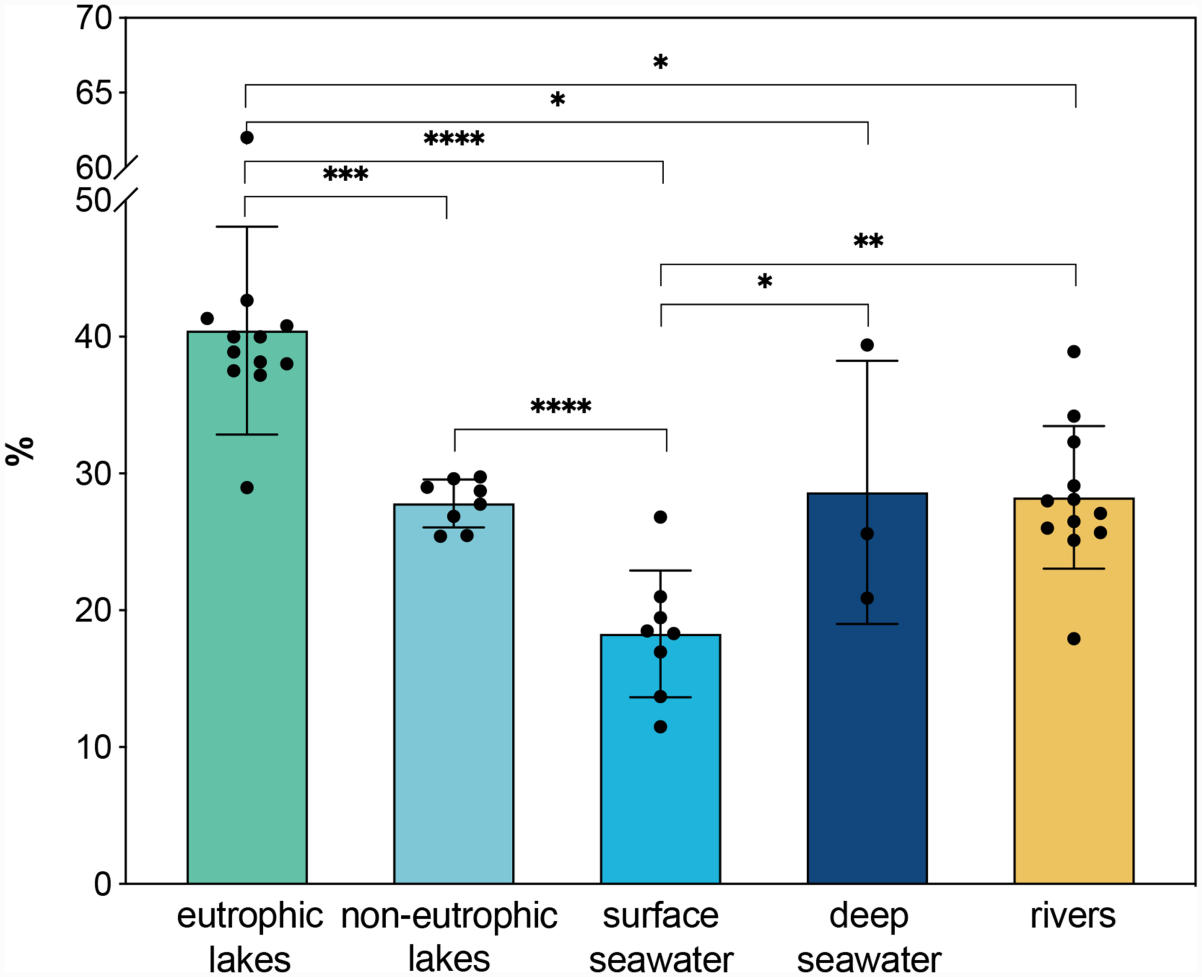
Comparison of relative abundance for CRAM across different aquatic environments via 1D ^1^H NMR. The data presented are compiled from multiple studies, including Kaiser et al. (2003), Woods et al. (2012), Li et al. (2016); Li et al. (2017, 2023), Goldberg et al. (2015), Lam and Simpson (2008), Lam et al. (2007), Zheng and Price (2012), Zheng et al. (2022), Hertkorn et al. (2006), Dittmar et al. (2008), and this study. *p < 0.05; **p < 0.01; ***p < 0.001; ****p < 0.0001.

The relative abundance of CRAM in eutrophic lakes, including Lake Taihu and Lake Ontario (Lam et al., 2007), was significantly higher (*p* < 0.05) than in most other aquatic environments (Figure 7). Elevated CRAM levels may result from both enhanced DOM supply and intensified microbial processing. Although surface seawater shows lower CRAM abundances than deep waters (*p* < 0.05) (Figure 7), CRAM quantification is complicated by contrasting DOC concentrations between water masses. Substantial variability also exists within river systems (Figure 7); for example, the Suwannee River exhibits high CRAM abundance (32–39%) attributed to strong humification (Lam and Simpson, 2009). Because few studies report SPE recovery rates, absolute CRAM stocks remain difficult to constrain; nonetheless, the combination of high DOC concentrations and high CRAM proportions suggests that eutrophic lakes contain substantially larger CRAM concentrations than many other aquatic environments. Indeed, a portion of the organic carbon fixed during cyanobacterial blooms is buried in sediments (Yu et al., 2022), while another fraction is transformed into RDOC which is stabilized and exported. In oligotrophic lakes, carbon storage often manifests as supersaturated CO_2_ due to relatively slow ecosystem metabolism, which is highly sensitive to temperature fluctuations (Cohen and Melack, 2020). In contrast, the widespread occurrence and chemical recalcitrance of CRAM across freshwater, estuarine, and marine systems suggest that RDOC in eutrophic lakes can persist over hydrological timescales and therefore represents a meaningful component of inland-water carbon sequestration.

Cyanobacterial blooms are expanding globally, currently affecting approximately 3.13 × 10^5^ km^2^ of lake area (Hou et al., 2022). Although the heterogeneity among global lakes, varying in depth, mixing regimes, bloom intensity, and hydraulic residence times, precludes a precise extrapolation of a global inventory from a single site, the mechanism observed in Lake Taihu suggests a widespread biogeochemical significance. The substantial accumulation of CRAM driven by bloom-associated microbial communities indicates that eutrophic lakes likely function as a significant, yet underquantified, reservoir of RDOC. Consequently, the interaction between cyanobacterial blooms and the MCP represents a critical pathway that underscores the potential importance of eutrophic lakes in regional and global carbon sequestration.

## 4 Conclusion

This study demonstrates that cyanobacterial blooms substantially enhance the formation of RDOC in eutrophic lakes through intensified microbial processing. Dominant bloom-associated bacterial taxa, including the *CL500-29 marine group*, the *hgc1 clade*, and *Sphaerotilus*, transform bloom-derived labile DOC into CRAM-rich RDOC, indicating that MCP-like mechanisms operate strongly in inland waters. The significant accumulation and persistence of CRAM in Lake Taihu, together with evidence from freshwater-marine continua, show that a considerable fraction of microbially processed RDOC can resist degradation over hydrological timescales and thus represents a long-lived carbon pool. As cyanobacterial blooms expand globally, RDOC production in eutrophic lakes may constitute a previously underrecognized component of the global carbon cycle. Incorporating RDOC dynamics into carbon budget assessments is therefore essential for accurately evaluating the long-term carbon sequestration potential of inland waters.

## Data Availability

The data presented in this study are publicly available. The data can be found here: https://www.ncbi.nlm.nih.gov/sra, accession numbers SRR23281899 - SRR23281909.

## Footnotes

1
https://openfluor.lablicate.com

2
https://www.i-sanger.com/

3
http://sthjt.jiangsu.gov.cn/
