# Supplementary material for: Accumulation of recalcitrant dissolved organic carbon during cyanobacterial blooms in Meiliang Bay, Lake Taihu: insights into the microbial carbon pump
Source: Front Microbiol. 2026 Feb 13;17:1753025. doi: 10.3389/fmicb.2026.1753025 (PMC12946145; doi:10.3389/fmicb.2026.1753025)
Supplement: Supplementary file 1 [file Supplementary_file_1.docx]

Supplementary Material

**Accumulation of Recalcitrant Dissolved Organic Carbon During Cyanobacterial Blooms in Meiliang Bay, Lake Taihu: Insights Into the Microbial Carbon Pump**

**Xiaohan Wu^1^, Xiaogang Chen^2^, Dan Wu^1^, Fenfen Zhang^1^*, Jinzhou Du^1^**

**^1^** State Key Laboratory of Estuarine and Coastal Research, East China Normal University, Shanghai 200333, China

**^2^** Key Laboratory of Coastal Environment and Resources of Zhejiang Province, School of Engineering, Westlake University, Hangzhou, 310024, China

**Corresponding Author***

Phone: 021-62232572

Email: [ffzhang@sklec.ecnu.edu.cn](mailto:ffzhang@sklec.ecnu.edu.cn)

# Contents

**Supplementary Figure S1.** Maps of the sampling sites in the Meiliang Bay of Taihu Lake.

**Supplementary Figure S2.** Photos of each sampling time.

**Supplementary Figure S3.** EEM contours of the three fluorescent components identified using EEM-PARAFAC.

**Supplementary Figure S4.** ^1^H NMR and DE-^1^H NMR spectra of the SPE-DOM from Meiliang Bay of Taihu Lake.

**Supplementary Figure S5.** Extended error bar plot showing the nine most abundant classes that differ significantly between the non-bloom group and the bloom group.

**Supplementary Figure S6.** Venn diagram of the numbers of OTUs common/unique between the non-bloom group and the bloom group.

**Supplementary Figure S7.** Heatmap of functional profiles of bacterial communities using FAPROTAX.

**Supplementary Table S1.** Basic physical and hydrological data in Taihu Lake.

**Supplementary Table S2.** Water parameters in Meiliang Bay.

**Supplementary Table S3.** Spectral characteristics of excitation and emission maxima of three fluorescent components identified by PARAFAC modeling, compared with previously identified sources.

**Supplementary Table S4.** Fluorescent components and optical properties in Meiliang Bay.

**Supplementary Table S5.** ^1^H NMR and DE-^1^H NMR section integrals for key substructures of the TH-DOM.

**Supplementary Table S6.** Diversity and richness parameters for IIIumina library.

**Supplementary Text S1.** CDOM and FDOM Parameter Calculations.

# Supplementary Figures

**Supplementary Figure S1.** Maps of the sampling sites in the Meiliang Bay of Taihu Lake.

**Supplementary Figure S2.** Photos of each sampling time.


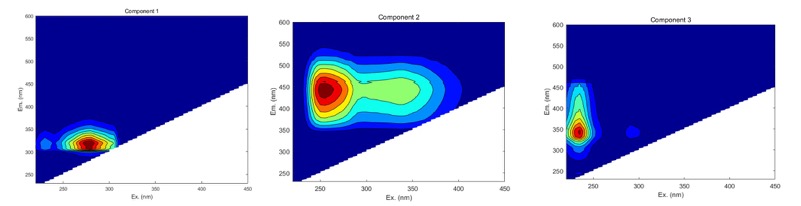


**Supplementary Figure S3.** EEM contours of the three fluorescent components identified using EEM-PARAFAC.

**Supplementary Figure S4.** ^1^H NMR (left) and DE-^1^H NMR (right) spectra of the SPE-DOM from Meiliang Bay of Taihu Lake. The peaks of D_2_O and CD_3_OD were clipped. DE = diffusion edited.

**Supplementary Figure S5.** Extended error bar plot showing the nine most abundant classes that differ significantly between the non-bloom group and the bloom group. Positive differences in mean relative abundance indicate classes overrepresented on the bloom group, while negative differences indicate greater abundance in the non-bloom group.

**Supplementary Figure S6.** Venn diagram of the numbers of OTUs common/unique between the non-bloom group and the bloom group.

**Supplementary Figure S7.** Heatmap of functional profiles of bacterial communities using FAPROTAX. X-axis indicates the sampling time during the one-year cyanobacterial bloom observation, and y-axis indicates representative functional groups.

# Supplementary Tables

**Supplementary Table S1.** Basic physical and hydrological data in Taihu Lake.

| Parameters | Data |
| --- | --- |
| Watershed area (km^2^) | 36500 |
| Lake surface area (km^2^) | 2338.1 |
| Mean depth (m) | 1.9 |
| water retention time (days) | 309 |
| Water storage (billion m^3^) | 4.4 |
| Runoff (billion m^3^) | 7.5 |
| Temperature (℃) | 0 ~ 38 ℃ |
| Mean temperature (℃) | 17.1 ℃ |
| Flood season | May - October |
| Dry season | November - April |
| Average rainfall (mm) | 1154 |
| Wind direction | Winter: northwester or wester |
|  | Summer: souther |
| Climate | Subtropics monsoon climate |

**Supplementary Table S2.** Water parameters in Meiliang Bay. DOC = dissolved organic carbon; Chl-α = Chlorophyll α.

| Sample | Sampling date | Temperature | pH | DOC | NO_3_^-^ | NO_2_^-^ | NH_4_^+^ | PO_4_^3-^ | *Chl-a* | Proportion of Cyanophyta in total phytoplankton |
| --- | --- | --- | --- | --- | --- | --- | --- | --- | --- | --- |
|  |  | (℃) |  | (μM C) | (μM) | (μM) | (μM) | (μM) | (μg L^-1^） | (%) |
| Jan | 2014/1/8 | 6.5 | 8.26 | 385.10 | 150.09 | 2.30 | 4.96 | 0.18 | 6.30 | 30 |
| Mar | 2014/3/16 | 14.5 | 7.79 | 382.82 | 134.79 | 0.64 | 2.29 | 0.10 | 28.78 | 5 |
| Apr | 2014/4/14 | 17.8 | 7.88 | 311.57 | 157.07 | 1.86 | 12.68 | 0.23 | 25.08 | 17 |
| May | 2014/5/20 | 22.5 | 8.34 | 354.68 | 233.09 | 3.04 | 2.33 | 0.35 | 26.23 | 82 |
| June | 2014/6/13 | 28.5 | 8.54 | 328.92 | 94.14 | 1.31 | 2.93 | 0.37 | 38.08 | 95 |
| July | 2014/7/14 | 29.0 | 8.66 | 330.20 | 83.24 | 3.18 | 0.55 | 0.41 | 44.50 | 98 |
| Aug | 2014/8/21 | 27.5 | 8.84 | 366.61 | 3.33 | 0.19 | 0.77 | 1.37 | 44.94 | 95 |
| Sep | 2014/9/27 | 25.0 | 7.74 | 391.40 | 20.23 | 2.64 | 4.13 | 2.21 | 34.10 | 92 |
| Oct | 2014/10/23 | 20.0 | 8.61 | 396.70 | 5.21 | 0.16 | 0.68 | 0.30 | 42.49 | 96 |
| Nov | 2014/11/14 | 13.3 | 8.12 | 348.29 | 3.84 | 0.29 | 0.78 | 0.97 | 28.20 | 84 |
| Dec | 2014/12/18 | 5.0 | 8.21 | 348.49 | 12.70 | 0.41 | 1.24 | 0.12 | 7.81 | 60 |
| Average and statistical differences (non-bloom versus bloom) | | | | | |  |  |  |  |  |
| non-bloom | | 13.3 | 8.10 | 356.53 | 137.55 | 1.65 | 4.70 | 0.19 | 18.84 | 39 |
| bloom |  | 23.9 | 8.42 | 360.35 | 35.00 | 1.30 | 1.64 | 0.94 | 38.72 | 93 |
| t-test |  | * | ns | ns | * | ns | ns | ns | ** | ** |

ns: not significant; *, ** and *** mean the correlation is significant at the 0.05, 0.01 and 0.001 level (2-tailed), respectively.

**Supplementary Table S3.** Spectral characteristics of excitation and emission maxima of three fluorescent components identified by PARAFAC modeling, compared with previously identified sources.

| Component | E*xmax*:E*m max* | Tradition Peak Coble (1996)；Coble et al.,(1998) | Comparison with other studies using PARAFAC | Description and probable source |
| --- | --- | --- | --- | --- |
| C1 | 280(230)/318 | Peak B: 275/305-310 | P5: 275/310 (Murphy et al., 2008) C8: 275/304 (Stedmon et al., 2005) C7: 270/299 (Yamashita et al., 2008) | Autochthonous tyrosine-like fluorescence |
| C2 | 255(335)/442 | Peak A: 230-260/380-460  Peak C: 320-360/420-480 | C3: 260 (370)/490 (Murphy et al., 2008) P8: <260 (355)/434 (Murphy et al., 2008)  C1: <260/458 (Yamashita et al., 2008) C1: <250 (355)/474 (Williams et al., 2010) | Terrestrial humic substances, widespread “A” and “C” peaks |
| C3 | 235/344 | Peak T: 225-230(275)/340-350 | C5: <250/370 (Williams et al., 2010) C6: 250 /356 (Kowalczuk et al., 2010) C7: 240 (300)/338 (Murphy et al., 2008) | Autochthonous tryptophan-like fluorescence. Amino acids, free or bound in proteins |

**Supplementary Table S4.** Fluorescent components and optical properties in Meiliang Bay. FI = fluorescence index; HIX = humification index; β:α = freshness index; SUVA_254_ = specific ultraviolet absorbance at 254 nm

| Sample | FI | HIX | β:α | SUVA_254_ | Fluorescent components (R.U.) | | |
| --- | --- | --- | --- | --- | --- | --- | --- |
|  |  |  |  | (L mg⁻¹ m⁻¹) | C1 | C2 | C3 |
| Jan | 1.75 | 0.60 | 1.01 | 1.94 | 2.13 | 0.49 | 1.75 |
| Mar | 1.75 | 0.46 | 1.09 | 1.95 | 2.27 | 0.40 | 1.69 |
| Apr | 1.78 | 0.48 | 1.07 | 2.39 | 2.08 | 0.40 | 1.59 |
| May | 1.83 | 0.67 | 1.00 | 2.11 | 1.80 | 0.54 | 1.52 |
| June | 1.78 | 0.72 | 1.02 | 2.27 | 1.45 | 0.50 | 1.54 |
| July | 1.77 | 0.69 | 1.00 | 2.26 | 1.41 | 0.48 | 1.64 |
| Aug | 1.84 | 0.99 | 0.89 | 2.04 | 1.08 | 0.59 | 1.53 |
| Sep | 1.81 | 1.05 | 0.91 | 1.90 | 1.02 | 0.67 | 1.55 |
| Oct | 1.80 | 1.27 | 0.90 | 1.90 | 0.99 | 0.70 | 1.63 |
| Nov | 1.80 | 0.81 | 0.97 | 2.15 | 1.26 | 0.51 | 1.58 |
| Dec | 1.78 | 0.77 | 0.99 | 2.14 | 1.28 | 0.47 | 1.50 |
| Average and statistical differences (non-bloom versus bloom) | | | |  |  |  |  |
| non-bloom | 1.78 | 0.59 | 1.03 | 2.11 | 1.91 | 0.46 | 1.61 |
| bloom | 1.80 | 0.77 | 0.98 | 2.16 | 1.47 | 0.53 | 1.56 |
| t-test | ns | ** | * | ns | *** | * | ns |

ns: not significant; *, ** and *** mean the correlation is significant at the 0.05, 0.01 and 0.001 level (2-tailed), respectively.

**Supplementary Table S5.** ^1^H NMR and DE-^1^H NMR section integrals for key substructures of the SPE-DOM from Lake Taihu. DE = diffusion edited; MDLT = material derived from linear terpenoids; CRAM = carboxyl-rich alicyclic molecules; carb = carbohydrates; arom = aromatic and phenolic constituents. The spectra were normalized to the identical total NMR integral.

| Sample | SPE-DOM recovery | ^1^H NMR constituents | | |  | DE-^1^H NMR constituents | | | |
| --- | --- | --- | --- | --- | --- | --- | --- | --- | --- |
|  |  | arom | carb | CRAM | MDLT | arom | carb | CRAM | MDLT |
|  | (%) | 8.4 - 6.5 | 4.5 - 3.48 | 3.17 - 1.6 | 1.6 - 0.6 | 8.4 - 6.5 | 4.5 - 3.48 | 3.17 - 1.6 | 1.6 - 0.6 |
| Jan | 51 | 3.8 | 18.8 | 37.5 | 36.3 | 3.4 | 20.2 | 36.0 | 37.1 |
| Mar | 40 | 4.3 | 18.8 | 29.0 | 46.4 | 3.5 | 17.6 | 25.9 | 51.1 |
| Apr | 38 | 2.8 | 18.1 | 38.9 | 40.3 | 1.1 | 19.5 | 33.3 | 42.5 |
| May | 45 | 4.2 | 18.3 | 38.0 | 38.0 | 2.4 | 19.0 | 35.7 | 40.5 |
| June | 45 | 1.3 | 13.3 | 41.3 | 44.0 | 3.4 | 17.2 | 36.8 | 36.8 |
| July | 46 | 2.7 | 14.7 | 40.0 | 41.3 | 4.6 | 19.5 | 35.6 | 35.6 |
| Aug | 44 | 3.9 | 15.8 | 38.2 | 38.2 | 4.4 | 18.7 | 36.3 | 35.2 |
| Sep | 45 | 2.6 | 15.8 | 40.8 | 36.8 | 4.4 | 18.7 | 37.4 | 34.1 |
| Oct | 47 | 1.3 | 16.0 | 42.7 | 38.7 | 3.7 | 20.0 | 37.7 | 33.5 |
| Nov | 50 | 2.7 | 16.0 | 40.0 | 38.7 | 3.3 | 17.8 | 37.8 | 35.6 |
| Dec | 49 | 2.6 | 12.8 | 37.2 | 46.2 | 3.3 | 17.6 | 35.2 | 40.7 |
| Average and statistical differences (non-bloom versus bloom) | | | | |  |  |  |  |  |
| non-bloom | 45 | 3.5 | 17.4 | 36.1 | 41.4 | 2.7 | 18.8 | 33.2 | 42.4 |
| bloom | 46 | 2.4 | 15.3 | 40.5 | 39.6 | 4.0 | 18.7 | 36.9 | 35.1 |
| t-test | ns | ns | ns | * | ns | ns | ns | * | ns |

ns: not significant; *, ** and *** mean the correlation is significant at the 0.05, 0.01 and 0.001 level (2-tailed), respectively.

**Supplementary Table S6.** Diversity and richness parameters for IIIumina library.

| Sample | 0.97 | | | | | |
| --- | --- | --- | --- | --- | --- | --- |
|  | Sequence number | ACE^a^ | chao^b^ | coverage | shannon | simpson |
| Jan | 51651 | 312.351611 | 307.923077 | 0.998816 | 3.635661 | 0.048538 |
| Mar | 58080 | 303.537406 | 305.357143 | 0.99889 | 3.378643 | 0.079692 |
| Apr | 57335 | 263.695406 | 281.882353 | 0.998866 | 3.377174 | 0.065127 |
| May | 55319 | 339.034341 | 337.885714 | 0.998545 | 3.345972 | 0.071745 |
| June | 58526 | 417.409517 | 382.857143 | 0.998027 | 3.023773 | 0.117232 |
| July | 58605 | 431.791108 | 455.088235 | 0.997953 | 3.805379 | 0.048347 |
| Aug | 61059 | 504.970513 | 486.671642 | 0.998101 | 4.302978 | 0.024402 |
| Sep | 66138 | 523.990759 | 508.850746 | 0.997707 | 4.111716 | 0.031521 |
| Oct | 68736 | 538.351231 | 533.241935 | 0.997559 | 3.918219 | 0.042359 |
| Nov | 54397 | 534.830518 | 516.558824 | 0.997312 | 3.376395 | 0.091663 |
| Dec | 49603 | 374.790723 | 385.617647 | 0.998052 | 3.30852 | 0.070011 |

^a^Abundance-based coverage estimator; ^b^Chao 1 species richness

# Supplementary Text

**CDOM and FDOM Parameter Calculations**

**SUVA_254_**

SUVA_254_ represents the specific ultraviolet absorbance at 254 nm and serves as an indicator of the aromaticity of DOM. Higher values denote a greater proportion of aromatic compounds. SUVA_254_ is typically calculated from the UV absorbance coefficient at 254 nm (α₂₅₄) and normalized to DOC concentration (Weishaar et al., 2003):

SUVA_254_ = *α*_254_/DOC

*α*_254_ = 2.303A/l

where A is the absorbance, l is the optical path length (m), and DOC is the concentration of dissolved organic carbon (mg L⁻¹).

**HIX (Humification Index)**

HIX represents the degree of DOM humification, with higher values indicating a higher extent of humification. HIX is calculated as the ratio of the integrated emission intensity from 435–480 nm to that from 300–345 nm under an excitation wavelength of 254 nm (Zsolnay et al., 1999). Ohno (2002) later modified the calculation to the following form:

HIX = ∑F(Ex=254nm, Em=435-480nm) / [ ∑F(Ex=254nm, Em=300-345nm) + ∑F(Ex=254nm, Em=435-480nm) ]

**FI (Fluorescence Index)**

FI is used to identify the source of DOM in natural waters (e.g., allochthonous vs. autochthonous). Lower FI values (~1.4) indicate degraded plant and soil organic matter, whereas higher values (~1.9) reflect DOM derived from bacterial and algal extracellular production. Cory et al. (2010) updated the original McKnight et al. (2001) definition. The FI is calculated as the ratio of emission intensities at 470 and 520 nm under an excitation wavelength of 370 nm:

FI = F(Ex=370nm, Em=470nm) / F(Ex=370nm, Em=520nm)

**BIX (Biological Index)**

BIX serves as an index of DOM origin, reflecting the contribution of recently produced DOM from planktonic or microbial sources. BIX values between 0.6 and 0.7 indicate a low contribution of autochthonous DOM, whereas values > 1 suggest a high contribution of recently produced microbial DOM (Huguet et al., 2009). BIX is calculated as the ratio of emission intensities at 380 and 430 nm under an excitation wavelength of 310 nm:

BIX = F(Ex=310nm, Em=380nm) / F(Ex=310nm, Em=430nm)

**β:α (Freshness Index)**

The β:α ratio represents the freshness of DOM, defined as the ratio of two known fluorescent components, where β corresponds to freshly produced DOM and α corresponds to highly decomposed DOM (Parlanti et al., 2000). The index is calculated as the emission intensity at 380 nm divided by the maximum emission intensity between 420 and 435 nm under an excitation wavelength of 310 nm (Miller et al., 2009):

β：α = F(Ex=310nm, Em=380nm) / MAX F(Ex=310nm, Em=420-435nm)

**CRAM Concentration Calculations**

^1^H NMR characterizes the relative distribution of non-exchangeable proton types across different functional group regions, providing structural information rather than direct quantification of carbon, and equating this directly to carbon distribution introduces uncertainty due to the varying H/C ratios of different DOM components (e.g., aliphatic vs. aromatic).

To address this and provide a more rigorous estimation, we have introduced an H/C correction factor derived from high-resolution mass spectrometry (FTICR-MS) data specific to CRAM and Lake Taihu DOM.

Instead of using generic marine values, we utilized data from our group's previous comprehensive investigation of Lake Taihu DOM (Zhang et al., 2014) to determine the specific H/C ratios for our correction:

CRAM H/C: The intensity-weighted average H/C ratio (H/Cwa) for CRAM components (CHO class molecules within the specific van Krevelen region) in Lake Taihu was found to range from 1.22 to 1.26. We selected the mean value of 1.24 for our calculation.

Bulk DOM H/C: The H/Cwa for the bulk DOM pool (including all identified elemental classes) in Lake Taihu ranged from 1.08 to 1.16. We selected the mean value of 1.12.

Application of the Correction: We calculated the correction factor to convert the proton-based fraction to the carbon-based fraction using the following equation:

Correction Factor = [H/Cwa(bulk DOM)]/[H/Cwa(CRAM)]

Substituting the values:

Correction Factor = 1.12/1.24 ≈ 0.90

Consequently, we have revised our estimations of CRAM concentration and the global RDOC stock by multiplying the original NMR-derived values by 0.90. This correction accounts for the fact that CRAM is relatively more hydrogen-rich than the average bulk DOM pool.

Uncertainty Analysis:

(1) H/C Conversion Strategy: We utilized the specific H/Cwa values for Lake Taihu derived from Zhang et al. (2014).

H/Cwa(CRAM): 1.22 - 1.26 (Mean: 1.24)

H/Cwa(bulk DOM): 1.08 - 1.16 (Mean: 1.12)

Calculation of the fluctuation range of the correction factor:

Scenario A: Minimum Discount (Maximum Correction Factor)

Assume Bulk DOM saturation is high (1.16), while CRAM saturation is relatively low (1.22).

Factor max = 1.16/1.22 ≈ 0.95

Scenario B: Maximum Discount (Minimum Correction Factor)

Assume Bulk DOM saturation is low (1.08), while CRAM saturation is high (1.26).

Factor min = 1.08/1.26 ≈ 0.86

Due to fluctuations in the H/C ratio, the Correction Factor for converting the NMR signal to carbon concentration ranges between 0.86 and 0.95.

(2) Sensitivity Analysis: To quantify the uncertainty requested by the reviewer, we calculated the propagation of errors from two sources:

Chemical Uncertainty: By permuting the upper and lower bounds of the H/C ratios (see above), the correction factor varies between 0.86 and 0.95, with an average value of 0.90.

Concentration Uncertainty: We incorporated the standard deviation of the measured CRAM concentrations.

# References

Weishaar, J. L., Aiken, G. R., Bergamaschi, B. A., Fram, M. S., Fujii, R. and Mopper, K. (2003). Evaluation of specific ultraviolet absorbance as an indicator of the chemical composition and reactivity of dissolved organic carbon. *Environmental Science & Technology* 37(20): 4702–4708. doi: 10.1021/es030360x

Zsolnay, A., Baigar, E., Jimenez, M., Steinweg, B. and Saccomandi, F. (1999). Differentiating with fluorescence spectroscopy the sources of dissolved organic matter in soils subjected to drying. *Chemosphere* *38*(1): 45-50. doi: 10.1016/S0045-6535(98)00166-0

Ohno, T. (2002). Fluorescence inner-filtering correction for determining the humification index of dissolved organic matter. *Environmental science & technology* *36*(4): 742-746. doi: 10.1021/es0155276

Cory, R.M., Miller, M.P., McKnight, D.M., Guerard, J.J. and Miller, P.L. (2010). Effect of instrument‐specific response on the analysis of fulvic acid fluorescence spectra. *Limnol. Oceanogr. Methods* 8, 67–78. doi: 10.4319/lom.2010.8.67

McKnight, D.M., Boyer, E.W., Westerhoff, P.K., Doran, P.T., Kulbe, T. and Andersen, D.T. (2001). Spectrofluorometric characterization of dissolved organic matter for indication of precursor organic material and aromaticity. *Limnology and Oceanography* *46*(1), 38-48. doi: 10.4319/lo.2001.46.1.0038

Hertkorn, N., Benner, R., Frommberger, M., Schmitt-Kopplin, P., Witt, M., Kaiser, K., et al. (2006). Characterization of a major refractory component of marine dissolved organic matter. *Geochim. Cosmochim. Acta* 70(12), 2990–3010. doi: 10.1016/j.gca.2006.03.021

Huguet, A., Vacher, L., Relexans, S., Saubusse, S., Froidefond, J.M. and Parlanti, E. (2009). Properties of fluorescent dissolved organic matter in the Gironde Estuary. *Organic Geochemistry* *40*(6), 706-719. doi: 10.1016/j.orggeochem.2009.03.002

Parlanti, E., Wörz, K., Geoffroy, L. and Lamotte, M. (2000). Dissolved organic matter fluorescence spectroscopy as a tool to estimate biological activity in a coastal zone submitted to anthropogenic inputs. *Organic geochemistry* *31*(12), 1765-1781. doi: 10.1016/S0146-6380(00)00124-8

Zhang, F., Harir, M., Moritz, F., Zhang, J., Witting, M., Wu, Y., et al. (2014). Molecular and structural characterization of dissolved organic matter during and post cyanobacterial bloom in Taihu by combination of NMR spectroscopy and FTICR mass spectrometry. *Water Res.* 57, 280–294. doi: 10.1016/j.watres.2014.03.064
